# High‐grade trochlear dysplasia increases patellofemoral joint pressure and decreases the knee extension torque, and tibial tubercle anteriorisation does not correct these effects: Biomechanical study in vitro

**DOI:** 10.1002/ksa.12570

**Published:** 2024-12-25

**Authors:** Michael Dan, Maria Moralidou, Isabelle Kuder, Richard J. van Arkel, David Dejour, Andrew A. Amis

**Affiliations:** ^1^ Lyon Ortho Clinic Lyon France; ^2^ Mechanical Engineering Department Biomechanics Group, Imperial College London UK

**Keywords:** knee extension torque, patellofemoral joint dysplasia, tibial tubercle anteriorisation, trochleoplasty

## Abstract

**Purpose:**

High‐grade femoral trochlear dysplasia is associated with anterior knee pain, patellar maltracking, instability and the development of osteoarthritis. Scientific studies have signified the importance of trochlear resection on the knee extensor mechanism, and dysplasia can be addressed by a groove‐deepening trochleoplasty. Alternatively, tibial tubercle anteriorisation has been proposed to reduce patellofemoral joint (PFJ) pressure and alleviate pain from osteoarthritis. However, the relative contributions of articular changes in the sagittal and axial planes remain unknown. This study aimed to better understand the effect of these different osteotomies, that alter the sagittal plane geometry, on PFJ biomechanics.

**Methods:**

Seven cadaveric knees were used to measure the following factors: (1) PFJ contact pressure; (2) Knee extension torque (KET); and (3) Patellar kinematics at 60°, 45°, 30°, 15° and 0° of knee flexion among four different osteotomy states: native, anteriorised trochlea, combined anteriorised trochlea and anteriorised tibial tubercle, and anteriorised tibial tubercle. Analysis was made using a two‐way repeated‐measures analysis of variance.

**Results:**

Anteriorising the trochlea increased mean PFJ contact pressures ×2.9 at 0° (*p* = 0.024) and ×2.2 (*p* = 0.029) at 15° flexion compared to the native state. Peak pressures increased ×4.9 at 0° and ×3.3 at 15° (n.s.). Anteriorising the trochlea reduced KET 18% (*p* = 0.001) at 40° flexion and 19% (*p* = 0.009) at 50°. The patella was anteriorised 8 mm in the extended knee (*p* < 0.001) and flexed 8° at 45° knee flexion (*p* < 0.001) compared to the native state. Elevating the tibial tubercle, alone or combined with an anteriorised trochlea, did not have a significant effect on the respective outcome measurements.

**Conclusion:**

An anteriorised trochlea elevated PFJ contact pressure, reduced KET and altered patellar position during knee flexion/extension movement, while a tibial tubercle anteriorisation had a negligible opposite effect. These findings indicate that symptoms associated with high grade trochlear dysplasia may be addressed better at the trochlea, rather than at the tibial tubercle.

**Abbreviations**
KET: knee extension torque
PF: patellofemoral
PFJ: patellofemoral joint

## INTRODUCTION

Abnormal morphology of the trochlear groove ‐ trochlear dysplasia—is present in 96% of patellofemoral joint (PFJ) instability, compared to 3% of normal controls [7]. Trochlear dysplasia results in the groove for the patella becoming flat or convex, and sometimes the anterior trochlea is more anteriorised than usual due to a positive offset from the femoral anterior cortex [7]. It is well known, both biomechanically and clinically, that the decreased congruence of the patella with the dysplastic trochlea leads to instability [1, 4], and is an aetiology for PFJ arthritis [18, 23, 31].

High‐grade trochlear dysplasia involves changes in both the axial and sagittal planes so that it is both flattened (or convex) transversely and more anteriorised, resulting in a lateral dislocation pattern [4, 6, 9]. However, the isolated influence of an anteriorised trochlea on PFJ reaction forces has not been investigated biomechanically, but it has been postulated that it will increase them [15]. A biomechanical study with a 3D printed trochlea simulating dysplasia has demonstrated increased PFJ reaction forces and contact pressure, but these models incorporated changes to both the axial and sagittal planes [33] while the present study aimed to understand the effects solely in the sagittal plane.

Maquet described a procedure of anteriorising the tibial tubercle to increase the knee extensor lever arm and reduce the PFJ reaction force [20]. More recently, Fulkerson advocated an ‘antero‐medialization osteotomy’ with elevation and medialisation of the tubercle. This incorporates the principles of the Maquet procedure to improve PF pain and arthritis and also realigns the patellar tendon medially [10, 11]. Rue et al. [27] reported that PFJ peak contact pressures were reduced by a mean of 26% at 30° flexion and 29% at 60° after a straight 10 mm anteriorisation of the tibial tubercle. Nakamura et al. [24] showed that anteriorisation of the tibial tubercle caused the patella to extend, moving the contact area to the proximal pole. If the anteriorisation exceeded 10 mm, the contact pressures were raised. However, these biomechanical studies did not involve a dysplastic trochlea. Rather than elevate the tibial tubercle, Goutallier et al. advocated deepening trochleoplasty to decrease the trochlear anterior offset, aiming to reduce PFJ reaction forces and PF pain [13, 14]. In PFJ arthroplasty, success is influenced by the ability to reduce the trochlear offset with respect to the anterior femoral cortex [2]. However, recessing the trochlea may detrimentally alter extensor mechanism torque by decreasing the moment arm [17]. Therefore, the sagittal position of the trochlea warrants further biomechanical analysis.

Clinically, there is increasing literature highlighting that differences in the position of the trochlea relative to the tibial tubercle in the sagittal plane are associated with PF instability, pain, and cartilage damage [19, 25, 30]. Therefore, anteriorising the tibial tubercle to address trochlear anatomical abnormality may theoretically improve clinical outcomes. However, given that altered sagittal tibial tubercle–trochlear groove distance was correlated with increasing trochlear dysplasia [30], it would seem logical to address the abnormality at the dysplastic trochlea. The relative effect of treating the abnormality at the tibial tubercle, via anteriorisation, versus at the trochlea, by a deepening trochleoplasty, is not known.

The aim of this biomechanical study was to document the changes in the PFJ mechanics caused by changes in the sagittal plane at the trochlea, reflecting the trochlear anteriorisation seen in high‐grade dysplasia, best described as Dejour grade B. It was hypothesised that an anteriorised trochlea would increase PFJ contact pressure and alter the PFJ kinematics and knee extension torque (KET). It was also hypothesised that the increased PFJ contact pressure could be reduced by anteriorising the tibial tubercle.

## METHODS

This work measured changes in the PFJ mechanics: patellar tracking, joint contact pressure and KET caused by simulated bony procedures in vitro: anteriorisation/recession of the anterior trochlea and anteriorisation of the tibial tubercle, both isolated and combined.

### Specimen preparation

Seven fresh‐frozen Caucasian cadaveric knees (mean age: 64 years, range 54–76, 4 M:3 F, 4 L:3 R) with no history of knee surgery or disease were sourced from an accredited tissue bank, following ethical approval from the local Research Ethics Committee. Power analyses based on prior studies [12, 28] showed that with seven specimens, differences of PF contact pressure of 0.3 MPa, of KET of 1.2 Nm and of patellar tracking of 6 mm (patellar lateral shift) could be identified with 80% power and 95% confidence. Specimens consisted of 250 mm of femur and tibia. They were stored in a freezer at −20°C before use and thawed overnight prior to the day of experimentation.

**Figure 1:**
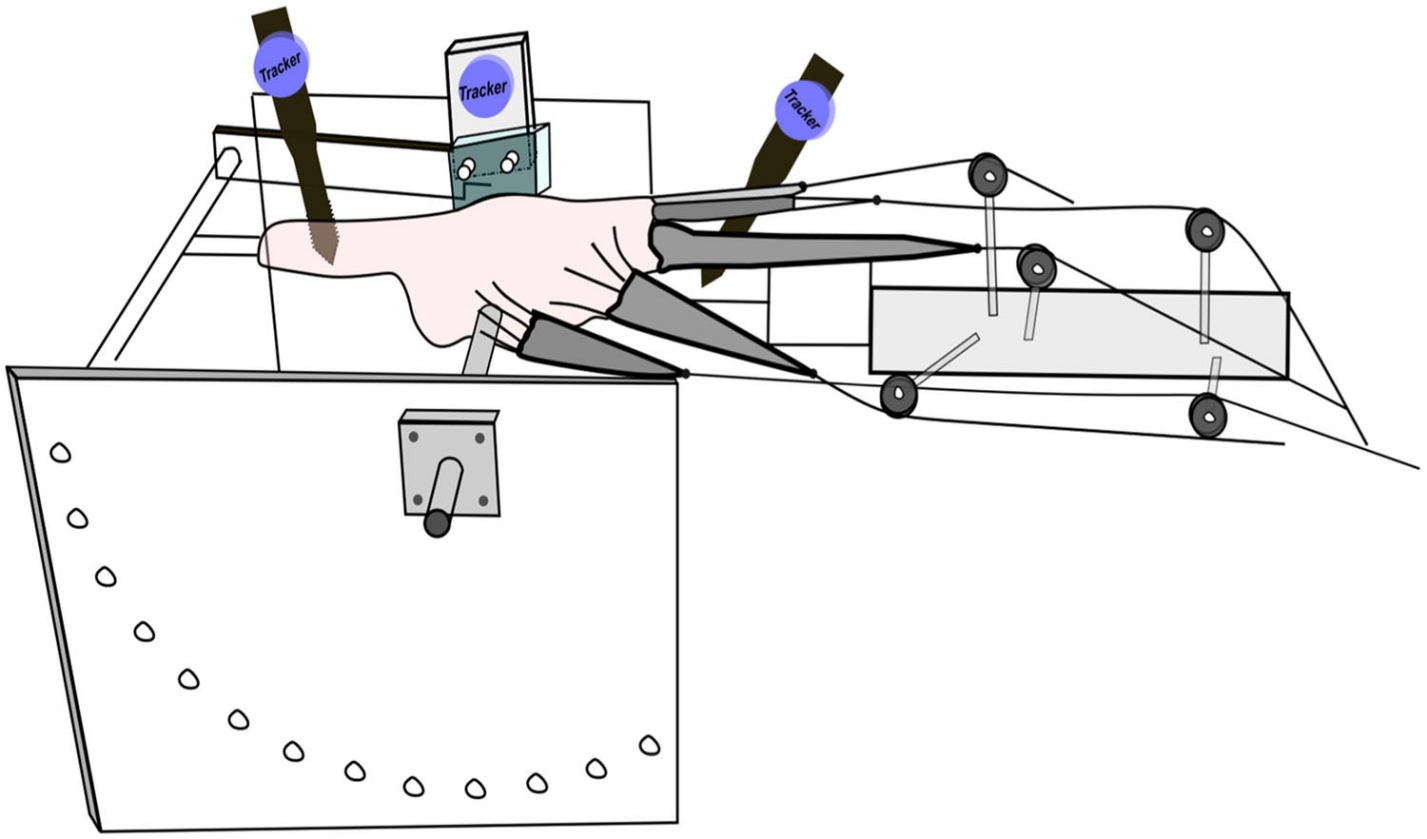
Testing setup demonstrating the femur potted, with the quadriceps muscles and tendons secured by cloth and rope and their loading cables led in physiological directions around pulleys and the tibia extended against a strain‐gauged restraining bar. Optical tracking targets are attached to each bone.

Removal of skin and subcutaneous fat enabled further individual dissection of the quadriceps tendons, the iliotibial band and hamstrings [12]. The dissected muscles and tendons were bound and stitched with cloth material to be attached to hanging weights via a pulley system, simulating physiological knee extension/flexion movement [17] (Figure 1). For this, a total force of 225 N was applied, as previously described [12]. Fixation of the fibula to the tibia via a transcortical screw prevented any potential nonphysiological movement due to the absence of the ankle [17].

### Testing apparatus

The proximal end of the femur was potted in a cylindrical tube using polymethylmethacrylate bone cement, aligned to the femoral anatomical axis [12]. The knee was mounted in the testing apparatus with the femoral transepicondylar axis aligned to the flexion‐extension axis of the knee extension rig by using guidewires inserted through the pivot bearings [12, 17] (Figure 1). A metal rod was inserted into the tibial distal canal, extending at least 100 mm, allowing control of the knee joint flexion/extension movement [12]. This method enabled restrained knee flexion/extension under physiologically distributed muscle loading to measure PFJ contact pressure, KET and patellar kinematics, using a repeated measures design.

### Surgical procedures

**Figure 2:**
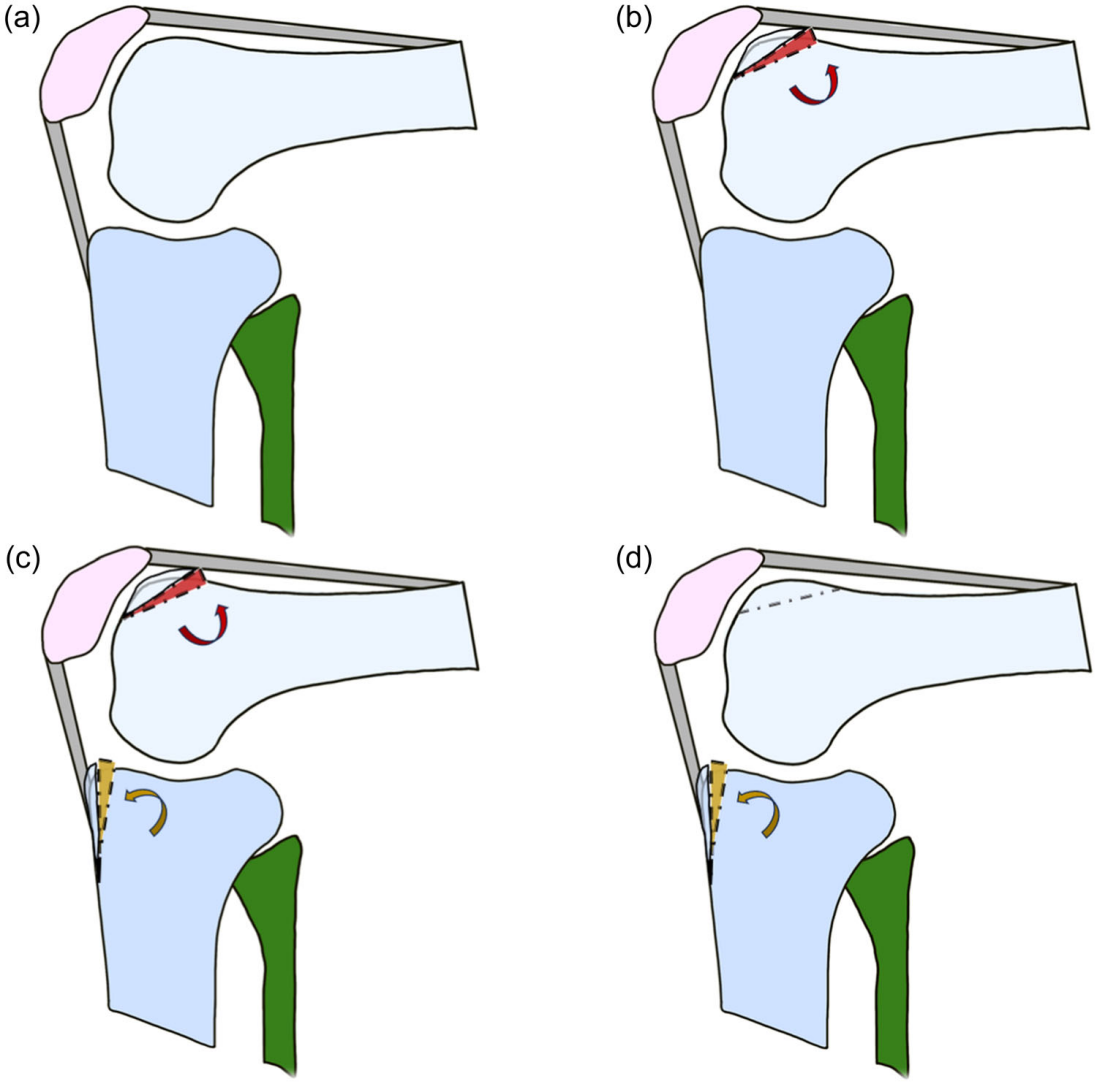
Osteotomies. (a) Native state; (b) Anteriorisation of the trochlea by 10 mm to simulate abnormal trochlear anatomy; (c) Combined anteriorisation of the trochlea and tibial tubercle, simulating both abnormal trochlear anatomy and 10 mm tibial tubercle anteriorisation surgery; (d) Isolated tibial tubercle anteriorisation with a 10 mm wedge, simulating tibial tubercle elevation surgery.

After reflecting the suprapatellar synovial pouch, the proximal edge of the trochlea was anteriorised by an osteotomy cut from proximal to distal, starting in line with the anterior cortex of the femur, leaving the distal articular cartilage intact to act as a hinge. The subarticular cancellous bone was left intact anterior to the osteotomy cut to preserve the axial plane morphology of the trochlear surface. A polyurethane foam (SawBone USA Cancellous replica) wedge was shaped so the proximal edge of the articular trochlea was anteriorised by 10 mm (Figure 2b). It was secured with a recessed AO cancellous screw proximally, and the suprapatellar synovium reflected back to cover it.

The tibial tubercle osteotomy was 60 mm long, leaving the distal hinge intact, and secured with a central large fragment AO screw. A triangular wedge was cut from polyurethane foam and inserted so that the insertion of the patellar tendon was anteriorised 10 mm (Figure 2c).

This allowed for four conditions to be tested

(1) Native state;
(2) Anteriorised trochlea;
(3) Anteriorised trochlea plus anteriorised tibial tubercle;
(4) Anteriorised tibial tubercle.

### Contact pressure measurements

Contact pressures between the patella and trochlea were measured using a Tekscan 5051 pressure sensor (Tekscan, I‐Scan) [29]. Before testing each knee specimen, each sensor was preconditioned and calibrated using a material testing machine (Instron 5565), according to the method recommended by the Tekscan company.

Care was taken to avoid disruption of the retinacula when inserting the sensor into the joint. With the proximal quadriceps elevated, two artery forceps were passed through portals adjacent to the patellar tendon to pass the sensor from proximal to distal through the suprapatellar pouch so that it covered the trochlea, before the rectus femoris was tensed to ensure the film was centred when compressed.

After loading all the muscles, pressure data were collected while locking the rig for 5 s intervals at 60°, 45°, 30°, 15° and 0° of knee flexion. Each test was repeated three times.

### KET measurement

The KET was measured by quantifying the force exerted by the metallic rod extending from the distal tibia against the movable crossbar of the knee extension rig [17, 28] (Figure 1). The bar was part of a strain‐gauged unit, aligned with the transepicondylar axis of each specimen. Measurements were taken between 0° and 90°, at 10° increments, using a data acquisition card (National Instruments) and repeated three times for each state.

### Optical tracking for patellar kinematics

Optical tracking measurements were taken using an active marker system (Optotrak Certus, NDI, Waterloo) [5]. Three trackers were rigidly attached to the femur, tibia and patella [5]. For the tibia and femur, bi‐cortical pins were used to attach the markers. For the patella, the tracker was rigidly fixed using a custom‐made, 3D‐printed polymeric block secured on its anterior surface using bone screws. A camera detecting these trackers was positioned to capture the whole knee specimen with all trackers visible throughout the range of motion. Digitisation of anatomical landmarks with respect to the trackers at full extension defined coordinate systems for each bone segment [16]. To ensure the repeatability of digitisation during different osteotomy states, these anatomical landmarks were marked with metallic screws before digitisation.

The anatomical landmarks on the femur were the medial and lateral epicondyles and the most proximal point on the anterior femur. For the tibia, the landmarks were the most proximal medial and lateral points and the most distal point on the anterior tibia. For the patella, four landmarks were used: the most medial and lateral points defined the mediolateral axis, the centre of which was defined as the centre of the patellar coordinate system, then the most proximal point on the anterior patellar flat surface and the insertion of the patellar tendon on the patellar anterior surface defined the proximodistal direction.

### Statistical analysis

Raw data were analysed and averaged in Matlab (2022, MathWorks) using custom‐written scripts, after which statistical analysis was performed in SPSS v.29 (IBM). A two‐way Repeated‐Measures Analysis of Variance (RMANOVA) was performed, with independent variables being the flexion angle (0°–90° at 10° increments) and the osteotomy state (e.g., normal state, anteriorised trochlea) after the assumption of data sphericity was checked using Mauchly's Test. In cases where the significance value was <0.05 or not displayed, the Greenhouse–Geisser correction was used if epsilon was <0.75; otherwise, the Huynd–Feldt correction was used.

First, the native state was compared with the state of an anteriorised trochlea to test whether an anteriorised trochlea would increase PFJ contact pressure and alter the PFJ KET and kinematics. Subsequently, the four different states were statistically compared to check if the increased PFJ contact pressure could be reduced by anteriorising the tibial tubercle. Post hoc paired *t* tests with Bonferroni correction were performed when statistical differences across groups were found. For the optical tracking data, due to the continuous nature of the measurements, one‐dimensional statistical parametric mapping (http://www.spm1d.org) using tests corresponding to an RMANOVA was utilised. The significance level was set at *p* < 0.05.

## RESULTS

### PFJ contact pressure

**Figure 3:**
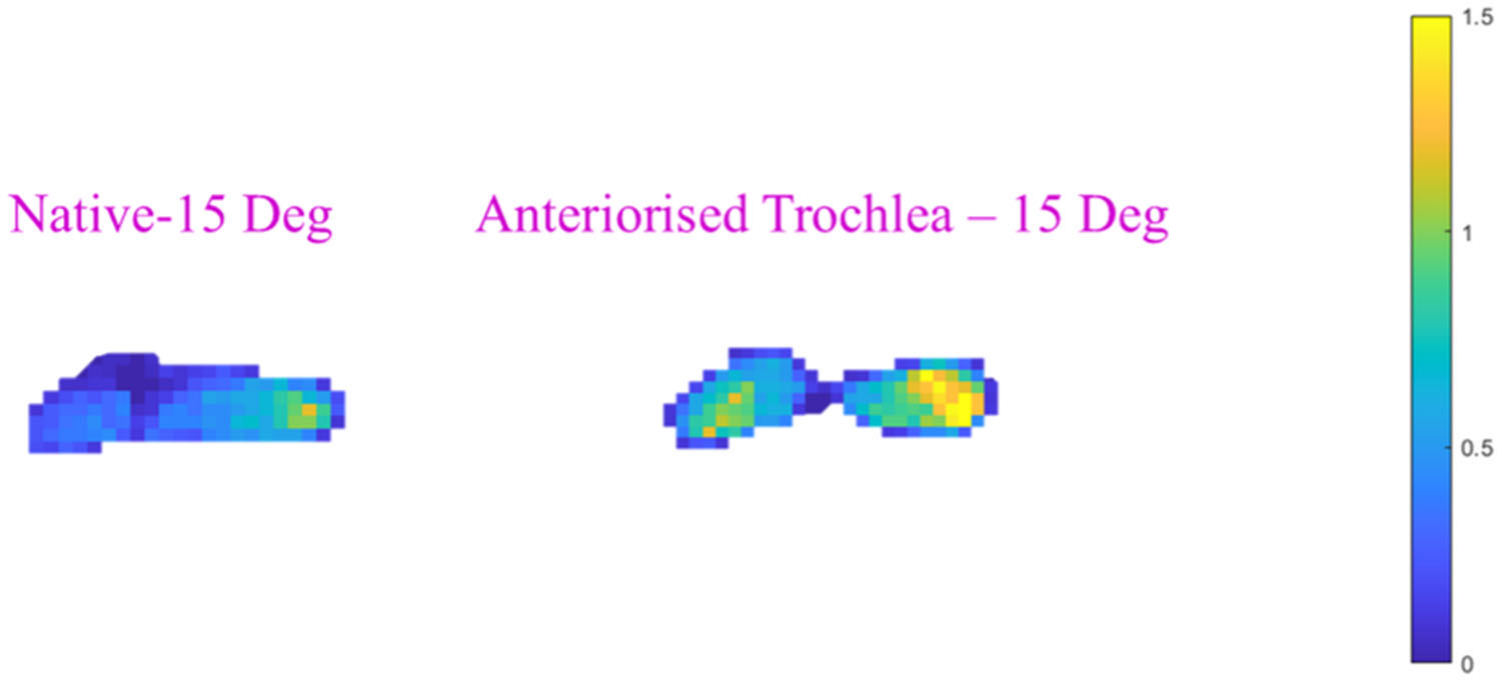
Typical TekScan pressure maps for the PFJ with the knee at 15° flexion with native trochlear geometry and anteriorised trochlea. The bar at the right side shows how the colour relates to the pressure, from 0 to 1.5 MPa.

**Figure 4:**
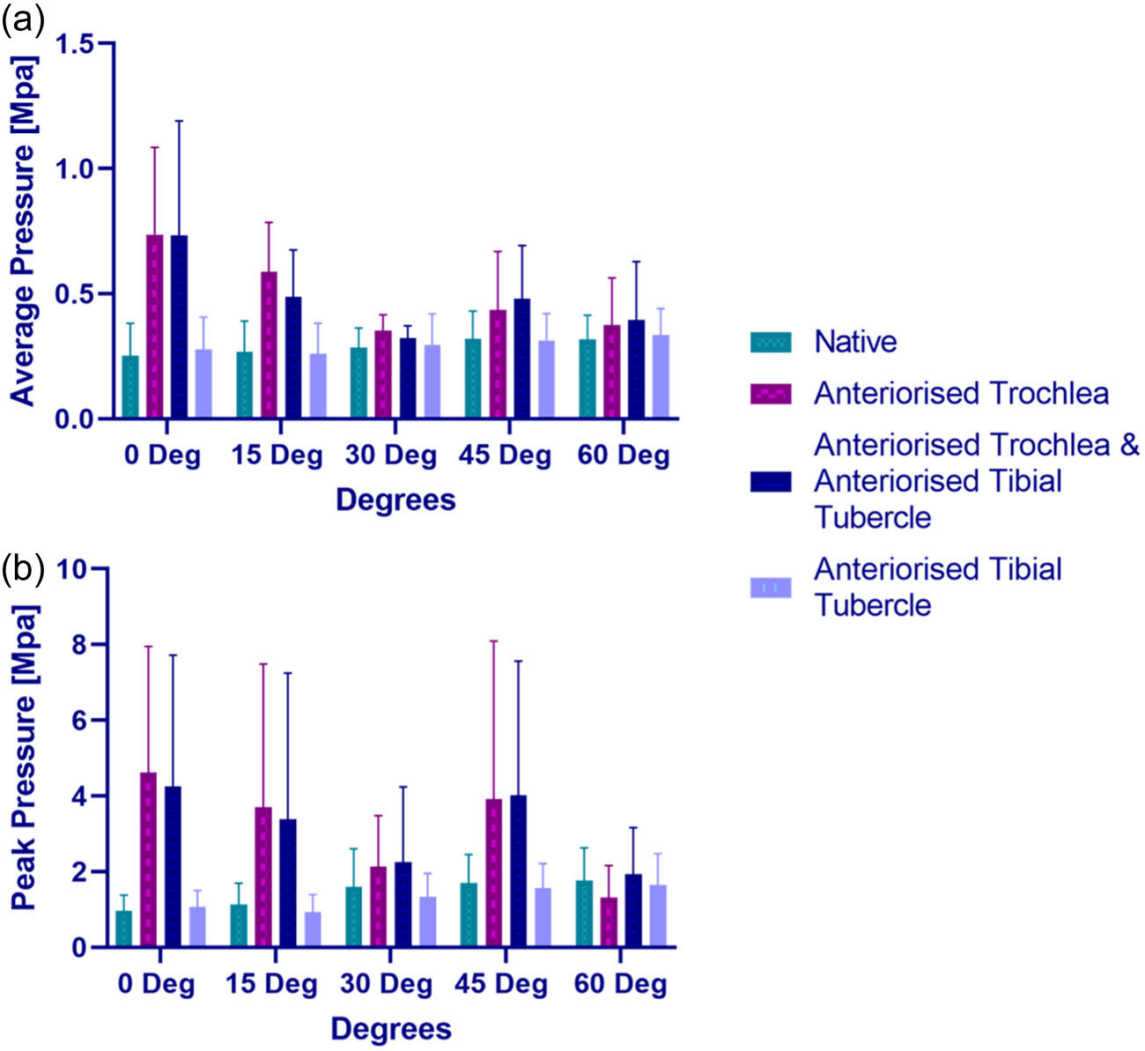
(a) Average contact pressures between 0° and 60° knee flexion, across the four different states of osteotomies at each angle of knee flexion. Anteriorising the trochlea increased mean PFJ contact pressures ×2.9 at 0° (*p* = 0.024) and ×2.2 (*p* = 0.029) at 15° flexion compared to the native state; (b) The 95% Quantile contact pressures between 0° and 60° knee flexion, across the four different states of osteotomies (*n* = 6). Peak pressures increased ×4.9 at 0° and ×3.3 at 15° (n.s.). Anteriorising the tibial tubercle did not change the contact pressure significantly when comparing either the anteriorised tubercle alone to the native knee or when comparing the anteriorised trochlea to the state with added tibial tubercle anteriorisation.

*Anteriorising the trochlea increased mean PFJ contact pressures ×2.9 at 0° (p* = *0.024) and ×2.2 (p* = *0.029) at 15° flexion compared to the native state* (Figures 3 and 4). Conversely, anteriorisation of the tibial tubercle had a negligible effect on the average contact pressure. An overall interaction effect was found (*p *= 0.037), but significant post hoc differences were not observed (Figure 4).

Regarding the peak PFJ contact pressures, a statistically significant difference was not found following either trochlear anteriorisation or tibial tubercle anteriorisation (Figure 4).

The corollary of these findings is that in the presence of dysplasia that has led to anterior prominence of the proximal edge of the trochlea, a 10 mm trochlear recession procedure back to native sagittal plane geometry could reduce the PFJ contact stresses by 66% at 0° knee flexion and by 53% at 15° flexion, while 10 mm anteriorisation of the tibial tubercle will not have a significant effect.

### KET

**Figure 5:**
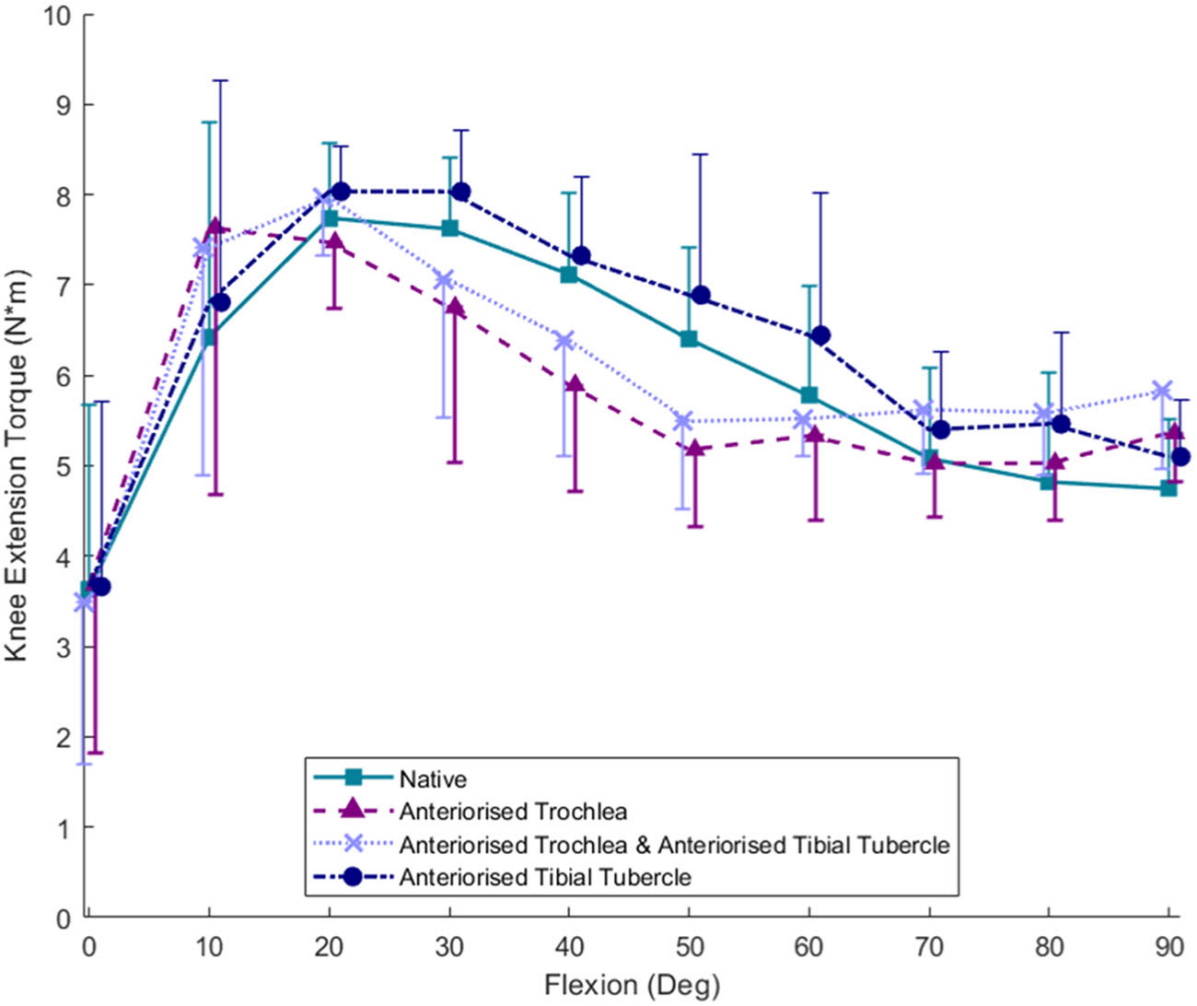
Knee Extension Torque (KET) (Nm) throughout the range of motion for the native, anteriorised trochlea, anteriorised trochlea plus anteriorised tibial tubercle and isolated anteriorised tibial tubercle states (*n* = 6). Anteriorising the trochlea reduced KET compared to the native state by 18% at 40° (*p* = 0.001) and 19% (*p* = 0.009) at 50° knee flexion. This reduction was slightly offset by anteriorising the tibial tubercle (*p* = 0.027). Isolated tibial tubercle anteriorisation did not increase the KET significantly above that of the native knee (*p* > 0.05).

An anteriorised trochlea resulted in less KET compared to the native state at 40° (*p* = 0.001)and 50° knee flexion (*p* = 0.009) (Figure 5). Adding an anteriorisation of the tibial tubercle to the knee with an anteriorised trochlea resulted in a small but statistically significant increase in mean KET compared to the anteriorised trochlear state (*p* = 0.027). However, although the isolated tibial tubercle anteriorisation tended to increase the KET above that of the native knee, the effect was not statistically significant.

### Patellar kinematics

Patellar kinematics are presented as changes from the native behaviour. Top row: the anteriorised trochlea lifted the patella anteriorly by 8 mm in the extended knee (*p* < 0.001). Middle row: anteriorisation of the tibial tubercle had little effect on patellar kinematics. Bottom row: (Left): The anteriorised trochlea caused the patella to be flexed by 8° near 50° knee flexion. (Centre): The patella was anteriorised 8 mm near to knee extension when the proximal edge of the trochlea was anteriorised. (right): It also led to a 2° lateral tilt near 50° knee flexion.

**Figure 6:**
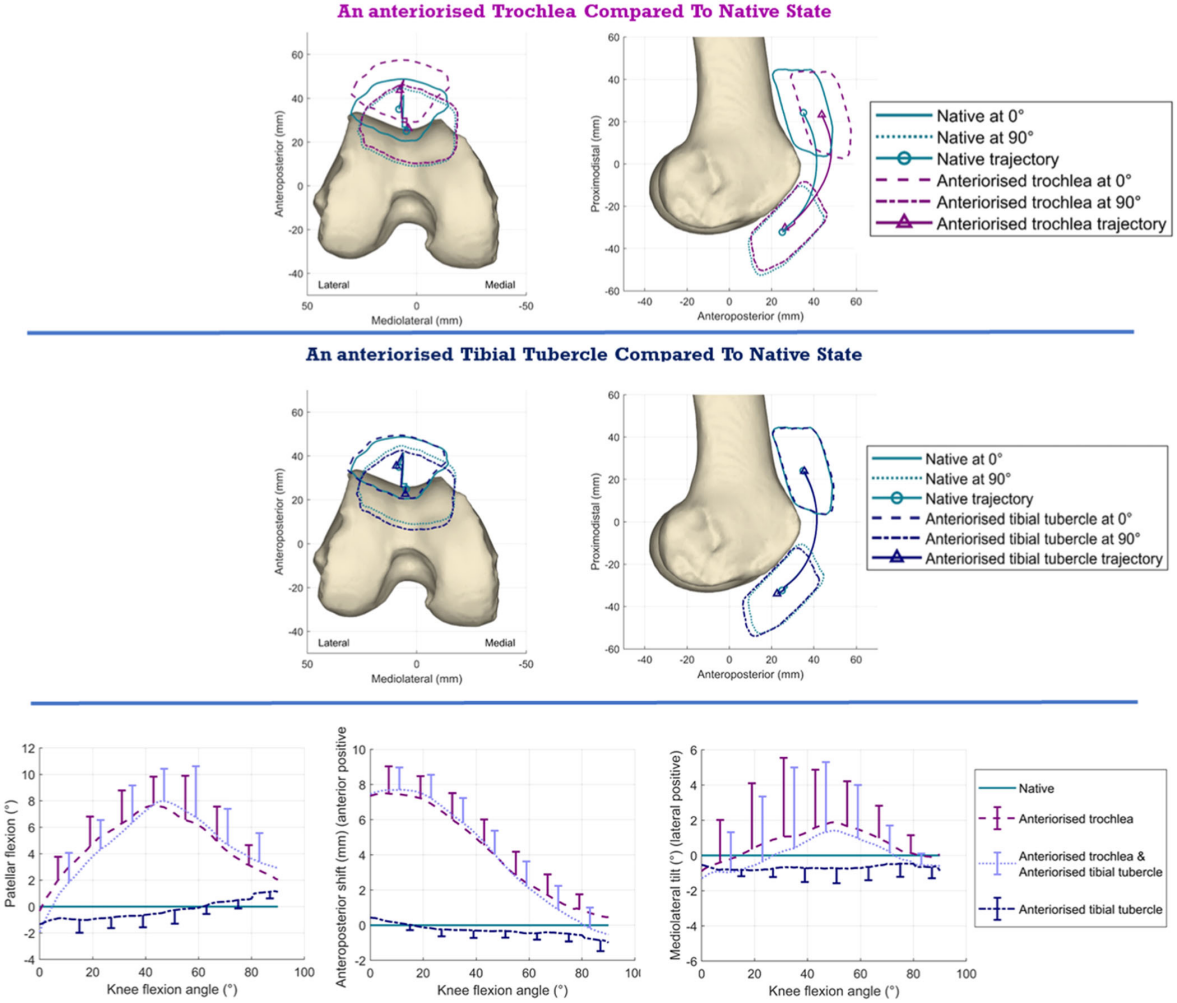
Changes of patellar kinematics away from native tracking in response to anteriorisation of the trochlea, anteriorisation of the tibial tubercle or both procedures.

Anteriorising the proximal edge of the trochlea lifted the patella anteriorly by 8 mm in the extended knee (*p* < 0.001; Figure 6). This anteriorisation of the path of the patella reduced to zero by 80° knee flexion. Tibial tubercle anteriorisation did not alter the patellar anterior position significantly (<1 mm) either in isolation or when added to the trochlear elevation.

A secondary effect was that the patella was flexed by up to 8° from native kinematics around 45° knee flexion (*p* < 0.001), due to it then resting on the distal‐facing slope of the proximally anteriorised trochlea (Figure 6). Tibial tubercle anteriorisation did not alter patellar flexion significantly (<1°), either in isolation or when added to the trochlear anteriorisation.

Anteriorising the trochlea did not affect the proximal‐distal position of the patella significantly. However, when the tibial tubercle was anteriorised, the patella was distalised by <2 mm (*p* = 0.016, 9°–85°) compared with the native position.

Patellar tracking was affected erratically among the knees, with a mean change of 2^o^ lateral tilt and 2 mm lateral translation around 50° knee flexion, but the variability meant that these mean changes were not significant. There were also small and erratic (<2°) changes in patellar rotation.

The overall picture is that the kinematic changes were almost entirely caused by trochlear anteriorisation. Tibial tubercle anteriorisation, either in isolation or combined with trochlear anteriorization, had only small effects (all <2 mm or <2°) across the arc of knee flexion.

## DISCUSSION

The most significant finding of this paper was that there were significant changes to the mechanics of the PFJ caused by anteriorising the proximal trochlea, simulating the abnormal geometry of the distal femur in the sagittal plane encountered with high‐grade dysplasia. The findings supported the hypothesis that an anteriorised trochlea would increase PFJ contact pressures but did not support the hypothesis that it would lead to increased KET. When compared to the effects of 10 mm trochlear anteriorisation, 10 mm tibial tubercle anteriorisation had little effect and the data do not support the hypothesis that this procedure would reduce the PFJ contact pressure. These findings suggest that abnormal biomechanics resulting from high‐grade trochlear dysplasia in the sagittal plane may be addressed better by trochlear recession rather than by tibial tubercle anteriorisation.

The articular contact pressures in the PFJ, and the effects of trochlear dysplasia, result from a combination of the overall geometry of the extensor mechanism in the sagittal plane and the effects on the congruency of the joint, such as increased sulcus angle, in the axial plane. While it has been postulated that an anteriorised trochlea will increase PFJ pressures [15], we are unaware of an investigation of the effects of trochlear anteriorisation without concomitant changes in the axial plane. The present study sought to separate these variables by only altering the geometry in the sagittal plane. An additional factor is that Maquet and Fulkerson advocated anteriorisation of the tibial tubercle as a means to reduce the PFJ force, a procedure that might act to counter the effects of trochlear anteriorization in the dysplastic knee [10, 11, 20]. Maugans et al. [21] investigated anteromedial tibial tubercle osteotomy and found that it reduced the force and contact pressure on the lateral facet of the trochlea and had little effect on the medial facet. That was as intended by Fulkerson, noting that articular lesions are most commonly on the lateral facet. This investigation has wider clinical relevance than trochlear dysplasia as there is debate regarding the optimal position of the trochlea in total knee replacement because of the effect on PFJ reaction forces. Some have advocated for an anteriorised trochlea to increase the extensor mechanism's lever arm [26], but outcomes of patellofemoral arthroplasty have been improved by recessing the trochlea to the anterior cortex [2]. The present study found elevated PFJ pressures for 0° and 15° knee flexion with an anteriorised trochlea, and this may play a role in the development of PFJ osteoarthritis.

The initial hypothesis that an anteriorised patella would lead to increased KET was based on the observation that it would elevate the line of action of the patellar tendon, hence increasing the extension moment about the knee flexion axis. However, the extensor moment decreased with anteriorisation of the trochlea at 40° and 50° knee flexion. Although not examined in detail, this likely reflects that the patella has to ‘climb uphill’ onto the anteriorised trochlea as the knee extends through this arc. Near to full extension, this effect was overcome and so the extension torque tended to be higher than with the native trochlear geometry. ‘Overstuffing’ the PFJ has deleterious effects on overall knee flexion [22] and may result in worse patient‐reported outcomes [3].

Trochlear anteriorisation caused significant changes in patellar kinematics in the sagittal plane. It had been expected that anteriorisation of the proximal trochlea would cause the patella to have an anteriorised path near knee extension, but it was also found that there was a significant patellar flexion around 45° knee flexion, reflecting that it was resting on the distal slope of the elevated portion of the trochlea. Patellar tracking, such as medial‐lateral translation (‘shift’) and tilt, was not affected significantly by trochlear elevation. Clinical studies have shown that patients with a trochlea with decreased depth have an associated lateral tilt and lateral translation of the patella. However, this decreased depth was also associated with an increased sulcus angle, so there was a combined anteriorised and flattened trochlea [8, 34]. The differing findings between the present study, which only examined the effects of changes in the sagittal plane, and these clinical studies suggest that patellar tilt and lateralisation may be more affected by axial plane changes in the trochlear geometry than sagittal plane changes.

Maquet hypothesised that the PFJ contact pressure could be reduced by anteriorising the tibial tubercle [20]. He advocated for over 25 mm of tibial tubercle elevation to reduce patellofemoral forces by 50%, but in clinical practice this led to problems with skin necrosis and aesthetic issues. The present study failed to show a significant reduction in PFJ contact stresses with 10 mm tibial tubercle anteriorisation, while others have calculated that it would reduce compression forces or the ratio between patellar compression force and quadriceps force by 20%–35% at 0° knee flexion and 10% at 60° [32]. Taken together, the results of this study suggest that in patients with trochlear dysplasia and patellofemoral pain or cartilage lesions, these are likely better addressed biomechanically at the trochlea, rather than at the tibial tubercle. Goutallier, Raou and Van Driessche [14] advocated a deepening trochleoplasty that would bring the trochlear groove back to the level of the anterior femoral cortex.

## LIMITATIONS

Although this study may have appeared to be limited by the number of specimens used, significant overall effects were found, so it was not underpowered. The main goal was to evaluate PFJ pressure changes with an anteriorised trochlea and/or tibial tubercle, which led to statistically significant changes. This was in keeping with the prospective power analysis, allowing minimisation of the number of specimens, in keeping with ethical principles related to cadaveric biomechanical studies. By working in vitro using fresh‐frozen specimens, the study was able to impose several operative procedures in each specimen, hence allowing a strong repeated‐measures data analysis.

## CONCLUSION

An anteriorised trochlea which represented high‐grade trochlear dysplasia (Dejour grades B&D) caused elevated PFJ pressures and led to decreased mid‐range KET. These effects were not offset by anteriorising the tibial tubercle. This suggests that high‐grade trochlear dysplasia is best addressed biomechanically at the trochlea with a trochleoplasty and not the tibial tubercle.

## AUTHOR CONTRIBUTIONS

**Michael Dan**: Study design; literature review; manuscript writing; data collection; manuscript editing. **Maria Moralidou**: Study design; literature review; manuscript writing; data collection; manuscript editing; statistical analysis. **Isabelle Kuder**: Statistical analysis; data collection; manuscript editing. **Richard J. van Arkel**: Supervision; study design; manuscript writing; manuscript editing. **David Dejour**: Study design; supervision; manuscript writing; manuscript editing. **Andrew A. Amis**: Supervision; study design; manuscript writing; manuscript editing. All authors read and approved the final manuscript.

## CONFLICT OF INTEREST STATEMENT

The authors declare no conflicts of interest.

## ETHICS STATEMENT

ICHTB HTA license 12275, REC approval 17/WA/0161, project R21053.

## Data Availability

Please contact the corresponding author for Data Availability.
